# Possibility to implement invasive species control in Swedish forests

**DOI:** 10.1007/s13280-015-0754-5

**Published:** 2016-01-07

**Authors:** Maria Pettersson, Caroline Strömberg, E. Carina H. Keskitalo

**Affiliations:** Department of Business Administration, Technology and Social Sciences, Luleå University of Technology, 971 87 Luleå, Sweden; Department of Geography and Economic History, Umeå University, 901 87 Umeå, Sweden

**Keywords:** Forestry, Invasive alien species, Sweden

## Abstract

Invasive alien species constitute an increasing risk to forestry, as indeed to natural systems in general. This study reviews the legislative framework governing invasive species in the EU and Sweden, drawing upon both a legal analysis and interviews with main national level agencies responsible for implementing this framework. The study concludes that EU and Sweden are limited in how well they can act on invasive species, in particular because of the weak interpretation of the precautionary principle in the World Trade Organisation and Sanitary and Phytosanitary agreements. In the Swedish case, this interpretation also conflicts with the stronger interpretation of the precautionary principle under the Swedish Environmental Code, which could in itself provide for stronger possibilities to act on invasive species.

## Introduction

Human pests and pathogens have spread across the world for hundreds of years; the successful ones slowly adapting to their new environments, sometimes contributing to the extinction of native species, reducing genetic variation and eroding gene pools, others being effectively eradicated by the new species (e.g. Hulme 2007; Vilà et al. 2010). However, human activities, economic globalisation, and more recently also climate change have seriously increased the movement potential for invasive alien species (IAS) to the point where biological invasions are considered one of the major threats to biodiversity (e.g. O’Brien and Leichenko 2000; Ricciardi 2006; COM 2011; Caffrey et al. 2014), especially in forests (Holmes et al. 2009). The damage caused by alien species is usually irreversible and difficult to predict because it occurs insidiously and involve novel interactions between species (e.g. Kumschick et al. 2015). Thus, while most non-indigenous potential pests are innocuous, some are directly harmful once introduced in a new environment, and some may prove hazardous, in which case the impact is difficult to measure (Holmes et al. 2009; Brunel et al. 2013).

Invasion by alien species may entail significant costs. The introduction of non-indigenous species, for example via international trade, may be considered a negative external effect in the sense that the risk for social and environmental damage, as a result of for example pest outbreaks, is not taken into account by the actors (Margolis et al. 2005; Perrings et al. 2010; Hantula et al. 2014). These indirect effects, or externalities, need to be reflected in the regulation of markets (Amitrajeet and Beladi 2006; Perrings et al. 2010), for example via legal or economic instruments. In the context of IAS management, several studies highlight the need for (additional) legislative action to adequately handle the issue of invasive alien species (Shine et al. 2000; Perrings et al. 2005; Caffrey et al. 2014). Smith et al. (2013) argue that “[a] strong strategic legislative framework is essential for addressing the complex challenges of invasive alien species”. In Sweden, the legal situation can be described as fragmented with numerous disconnected rules, which in combination with a lack of coordination between the responsible authorities has seriously hampered the control efforts (Pettersson and Keskitalo 2012). The Swedish Environmental Protection Agency suggested an investigation of the possibility to supplement existing regulations to cover all handling of invasive species (SEPA 2008). Several studies also emphasise the need for a more uniform interpretation and application of the precautionary principle (Pettersson and Keskitalo 2012; Keskitalo and Pettersson 2016). The issue of legislative action has been on the agenda also in the EU in recent years (e.g. COM 2008, 2011); even though the issue has been addressed in several legal acts, most invasive species have not been targeted by existing EU law. In 2013, the deliberations resulted in a proposal for an EU regulation on invasive alien species (COM 2013). The Regulation (EU) No 1143/2014, adopted by the Council on 29 September 2014, addresses the problem of invasive species in a comprehensive manner with the aim of protecting biodiversity and ecosystem services, and to mitigate social and economic impacts of biological invasions.

Although it has become increasingly important to prevent international movement of IAS and enhance rapid detection at borders, the rules pursuant to international trade regimes—such as the World Trade Organisation (WTO) and the North American Free Trade Agreement (NAFTA)—pose limitations to individual states’ as well as the EU’s, possibilities to impose restrictions on trade (Margolis et al. 2005). As the WTO sanitary and phytosanitary (SPS) agreement basically requires that the risks for harm are well documented and based on scientific proof for restrictions to be allowed, a comprehensive legal framework where the precautionary principle—to limit the risk of harm—plays a determining role may prove difficult to achieve (Pettersson and Keskitalo 2012). IAS are also addressed under the Convention on Biological Diversity (CBD); according to article 8(h) “each Contracting Party shall, as far as possible and as appropriate, prevent the introduction of, control or eradicate those alien species which threaten ecosystems, habitats or species”. In 2010, twenty biodiversity-related targets to be achieved within a decade were agreed upon by the contracting parties (the so-called Aichi Targets). With the strategic goal of reducing the direct pressures on biodiversity and promote sustainable use, Target 9 is specifically aimed at IAS: “By 2020, invasive alien species and pathways are identified and prioritized, priority species are controlled or eradicated, and measures are in place to manage pathways to prevent their introduction and establishment”. The Aichi Targets and the Strategic Plan are intended to be used as a flexible framework for the development of national and regional targets.^1^

The question of how biological invasions should be handled legally on different levels—from international agreements to national law and policy—is thus highly relevant. This study targets the Swedish institutional system’s capacity to handle biological invasion in the form of pathogens, plant and tree species both at present and under increased pressure of climate change and globalisation. The study takes into account what are seen as legal principles, specifically the precautionary principle and the polluter pays principle. The precautionary principle implies that action should be taken already when there is risk for something being harmful, even if this has not yet been proved. However, this principle may conflict with other principles, such as that of free trade, or less strict versions of the precautionary principle itself, such as under the SPS Agreement, which requires clear evidence of negative impacts to allow for restrictions in trade in a substance or material. The Polluter Pays Principle primarily aims at internalising negative external effects of economic activities, as is expressed in e.g. article 16 in the Rio Declaration, and implies that the polluter should bear the costs of carrying out the pollution prevention and control measures necessary to ensure that the environment is in an acceptable state (e.g. Government Offices of Sweden, prop. 1997/87:45). The aim of the paper is to identify and assess the function of the current regulatory framework as a mean to control external effects resulting from the introduction and spread of forest related pathogens, plant and tree species in Sweden. This study thus adds to Klapwijk et al. (2016) by highlighting the specific legal requirements and the way in which these are perceived as manageable or sufficient in the Swedish context.

## Materials and methods

The study is based on a legal review of the regulatory frameworks for plant and wood products, which determines applicable law, as well as semi-structured interviews with those involved in implementing this law.

With regard to the legislative study, the method of *constructive analytical jurisprudence* was used to analyse the concepts, rules and structures of the relevant laws. In this context, *constructive*—as opposed to dogmatic—is taken to mean “problem oriented”. This essentially implies approaching and analysing the legal framework with starting point in an actual problem rather than merely the linguistic and logical elucidation of legal concepts (Westberg 1992; Agell 1997). The identified problem in this case is the threat to forests represented by invasive alien species and pathogens. It is hence not only the interplay between rules and their position in the legal system that is being considered, but the social and political function of the rules as well. In the results section, the legal framework for control of invasive alien species in general, and plant and plant pests in particular, is outlined with regard to several levels of regulation that fundamentally impacts Sweden. This includes the international trade framework, the framework of EU law, and the national legal framework. The account includes both “hard law”, here defined from the perspective of its effectiveness at the stage of implementation^2^; i.e. the power to impose real obligations on the parties, and more “soft law” based agreements, which strength lie mainly in the parties’ willingness to abide by the agreement (e.g. Boyle 1999; Shaffer and Pollack 2010).

With regard to the interview study, persons were strategically identified and selected as responsible for the implementation of the law on the government level. Thus, semi-structured interviews were conducted with two persons from the Swedish Board of Agriculture, two persons from the Swedish Forest Agency, one person from the Swedish Environmental Protection Agency, and one person from the Ministry of Enterprise and Innovation at Government Offices of Sweden during the fall of 2014 and the spring of 2015. Each interview lasted for approximately 1 hour and 15 min, and interviews were recorded and transcribed in their entirety. The interview guide as well as thematic coding of the interviews focused on the framework for IAS and plant pests control outlined in this article.

## Results

### Legal framework: The international trade regime

Given that international trade is one of the most important sources to biological invasions, legal aspects of the problem must first be sought in the international trade regime which primarily is governed by the WTO. The WTO was established in 1994 and set to administer the agreements negotiated under the Uruguay Round (1986–1994) and to serve as a forum for future negotiations. The main task for the organisation is to supervise and liberate/facilitate international trade, e.g. by controlling trade barriers. The trade rules under the WTO regime are made up of several agreements covering goods, services and intellectual property. The agreements consist of six main parts, for which the multilateral General Agreement on Tariffs and Trade (GATT 1994) serves as the umbrella. For the issue of IAS, the GATT and the SPS Agreement are the most relevant of the six agreements.

One basic notion under the WTO regime is to remove barriers to trade by eliminating discrimination. Two main principles are set out to underpin this: (a) most-favoured-nation (MFN) treatment (Art. I, GATT), which implies that countries should give all their trading partners equal status as ‘most favoured nation’ and thus extend all countries the same trade preferences; and (b) national treatment (Art. III, GATT), which means that countries should treat its own and foreign products and services equally.

In relation to the GATT, the SPS Agreement *reaffirms* that while “no Member should be prevented from adopting or enforcing measures necessary to protect human, animal or plant life or health”, such measures must not constitute a means of “arbitrary or unjustifiable discrimination or a disguised restriction on international trade” (preamble). The agreement thus establishes rights as well as obligations; the right to take legal protective measures and the obligation to do so without creating unnecessary trade barriers. The rules of the agreement (most notably articles 2, 2.2, 3 and 5.1) focus on the obligations and contain provisions on how protective measures should be designed to not create trade barriers that are more intrusive than necessary to achieve the purpose (i.e. to protect e.g. human or plant health). Therefore, all SPS measures must be based on scientific principles and not be maintained without sufficient scientific evidence. The measures shall be preceded by a risk assessment, as appropriate to the circumstances, and as far as possible be based on international standards, guidelines and recommendations. Substance or material that could be seen as a risk of causing harm can thus only under certain conditions be limited prior to the presence of scientific evidence. This means that time can lapse before such studies have been undertaken, during which spread and novel interaction between invasive and native species may take place. Fundamentally, this framework limits the potential to implement a strong version of the precautionary principle.

WTO compatible legal standards for the control of plant products and pests are developed within the framework of the International Plant Protection Convention (IPPC), which does not supersede but rather enforce the limitations described above. The aim of this multilateral treaty is to protect cultivated and wild plants by preventing the introduction and spread of pests, and the substance of both the GATT and the SPS Agreement are recognised in the preamble to the IPPC. This is also reflected in the principles on which the convention is based, for example that phytosanitary measures shall: (a) only be applied when necessary; (b) be applied equally to countries of equivalent plant health and for the same pests; (c) must be published and motivated; and (d) imply least possible impact on international trade (MacLeod et al. 2010). The standards (ISPMs) set out in the IPPC aim to reduce the spread of pests and facilitate trade and include guidelines for pest risk analysis, surveillance and pest eradication; code of conduct for the import and release of biological agents; and requirements for the establishment of pest-free areas. Since the SPS Agreement recognises the IPPC as standard setting authority, WTO members are expected to base their phytosanitary measures on the standards established under the convention. However, while the IPPC in itself is binding on the contracting parties, the standards set out under the convention are not; they are subject to various interpretations, and occasionally, disputes, which ultimately have to be solved by the WTO. South Africa vs. EU is an example of an ongoing conflict. Here South Africa claims that the strength of the phytosanitary measures required by the EU are inconsistent with the level of risk posed by the introduction of Citrus Black Spot (CBS—caused by the fungus *Guignardia citricarpa*) on fruit that is imported into the EU (www.ippc.int).^3^

With regard to perceptions of implementation of this complex legal situation, all interviewees in the Swedish case noted that Sweden encourages free trade and does not question the principles of the SPS Agreement. However, it was also pointed out that the SPS Agreement limits the ability to prevent the introduction and spread of IAS, since many measures that need to be taken entail restrictions in trade. It was also noted that while free trade has a high value, having a system that fails to prevent pests or IAS from spreading will lead to high costs for measures ex post. Even if restrictions on trade may be costly for businesses, it may also increase competitiveness due to a high plant health status.

When asked about the possibility to implement the precautionary principle, all interviewees raised the issue of the different possible interpretations of the principle. Several interviewees stressed that applying a strong version of the precautionary principle, i.e. one that requires less evidence than the SPS Agreement, would be good for plant protection, and useful in uncertain cases when there could be serious and irreversible consequences of introduction and spread of species. Arguments against a strong version of the precautionary principle were also presented; for example that it can be used e.g. to protect one’s own business from competition, and that because measures are expensive to take, the decisions to take them should be well-grounded.

### EU law

Legal protection against introduction of species harmful to plant or plant products in the EU is provided by Directive 2000/29/EC; the so called Plant Health Directive. The Directive is a consolidated version of the 1976 Plant Health Directive (77/93/EEC), including subsequent amendments to that legislation, and also reflects international trade agreements by being compatible with the SPS Agreement. The current EU plant health regime is a complex system that builds on the original intra-community trade, as well as Third Country imports of plant and plant products, which was rebuilt in the early 1990s to create a single EU market (MacLeod et al. 2010). The main feature of the system is the legal space created to prevent entry and spread of foreign pests by means of the legal instruments: prohibition/banning and certification. The regime is based on the listing of harmful organisms into different categories, from particularly harmful organisms whose introduction and spread must be banned by all member states, to the listing of plants and plant products which must be subject to a plant health inspection, including special rules for protected zones (Annex I-VI, Directive 2000/29/EC).

The design of the EU plant health regime, under which movement into and within the Union, is basically allowed provided that the explicit restrictions and requirements are complied with, thus emphasises the importance of supporting free trade. The system, however, has had significant drawbacks, most prominently regarding its inability to control the increasing influx of harmful organisms as a result of globalisation of trade due to amongst other insufficient focus on prevention in relation to increased imports of high-risk commodities (COM 2013). The European Commission has therefore submitted a proposal for a new Regulation concerning protective measures against plant pests (COM 2013). The proposal contains potentially important differences compared to Directive 2000/29/EC. Schematically, pests are divided into three categories under the proposed Regulation: non-listed pests, quality pests and quarantine pests, where the latter is the main target for the Regulation. In addition to the implementing acts, member states are given some leeway in terms of possibilities of adopting additional or stricter measures. In order to ensure effective action against pests that are not qualified as Union quarantine pests, member states may take protective measures against the pests if they consider the criteria for EU quarantine pests fulfilled. Provided that it does not conflict with the free movement of e.g. plants member states will also “be allowed to adopt more stringent eradication measures than required by Union legislation” (COM 2013). Furthermore, the proposed Regulation obliges anybody who is aware of the presence of a quarantine pest to notify the authorities; it encourages member states to conduct surveys for the presence of pests; and it sets out eradication measures, including area restrictions, as well as rules for the establishment of contingency and eradication plans. All in all, it appears as if the proposed Regulation offers a more nuanced regulatory framework, where precautionary measures are at least supposed to play a bigger role. It is however difficult to assess the full consequences of the proposal at this stage.

To approach the problem of invasive species in a more holistic and coherent manner, a Regulation on invasive alien species was adopted in 2014. The primary objective of the regulation is to “prevent, minimise and mitigate the adverse effects of invasive alien species on biodiversity and ecosystem services, as well as to reduce their economic and social impact”. As a first step to achieve the objective, a list of IAS that pose a particular threat to the Union shall be drafted. To qualify as being of Union concern, the damage caused by the species should be significant enough to justify the adoption of dedicated measures. This, in turn, is assessed on the basis of certain criteria, all of which are in line with the SPS Agreement and include risk assessments. Furthermore, since prevention is preferable to reaction it is considered necessary that the list of species is continuously revised and updated. In case of a sudden and unexpected appearance of species that have not yet been defined as IAS, but where there is scientific evidence of its harmfulness, it will be possible for member states to adopt certain emergency measures. To otherwise be able to take more stringent and proactive (national) measures on non-listed species, special authorisation will be required. Legally, it will still be difficult for member states to implement certain prevention or protection measures since, as enforced in this proposal; member states cannot take action contrary to the Regulation (Keskitalo and Pettersson 2016).

### Possibilities of proactive measures

With regard to the possibilities to take proactive measures under this legal framework, several interviewees questioned the effectiveness of the new IAS Regulation since the principle of free trade will continue to limit the possibilities to prevent the introduction and spread of IAS. A general need for a clarification regarding the responsibilities of different authorities was also stressed, as there are invasive species that do not fall within the scope of any of the applicable legislations, implying that no one has the authority to take measures against such species. Furthermore, the distribution of responsibilities was described as unclear, which was perceived as problematic, for example if emergency measures are necessary. It was noted that the distribution of responsibility would be clearer with increased collaboration between the authorities, but that diverse approaches, priorities, definitions and interests of the different authorities had complicated collaboration in the past. Increased communication and the development of a more “unified voice” was therefore considered essential to achieve the kind of collaboration that will be required under the new IAS Regulation. It was also suggested that the Swedish government should establish a formal collaboration group, since setting aside resources and time would facilitate prioritisation of the work; if collaboration is supposed to be “squeezed into the daily work” it may not happen because of the time pressure.

One of the interviewees also predicted financial difficulties in complying with the new rules as it will be costly and funding will not be provided from the EU. However, several other interviewees pointed out that since the Regulation applies directly, the Swedish government has no choice but to allocate resources, The plant health regime was described as providing more developed tools than the IAS area, and it was suggested that some of the experience from this area could be used in the work with IAS.

Both with regard to plant pests and IAS, all interviewees stressed the need for more preventive measures, especially regarding IAS since they are usually not discovered until after the damage is done. A monitoring system for detection of new species was considered an important measure in this context, but the lack of continuity of funding was seen as an obstacle to the development of a stable monitoring system. Regarding plant pests, the previous EU legislation was considered to lack focus on prevention, and a monitoring system for proper surveillance of the Swedish territory was suggested, which is in line with the proposed EU Regulation.

The interviewees considered plant passports, as well as phytosanitary certificates (*sundhetsinty*g), to be strong and important tools, although the limited possibility to control if they are actually followed was pointed out as an issue. Other concerns raised in relation to plant passports were uncertainties regarding their design, on which conditions they should be issued, and how to recognise them. All of these things were expected to become clearer with the new Regulation. The fact that the plant health Regulation will not include invasive alien *plants,* which on EU level instead will be covered by the IAS Regulation, was however seen as an issue. Since invasive alien plants are regulated under the IPPC, which implementation is the responsibility of the Board of Agriculture, the EU legal framework will deviate from the systematics on international and national level. This discrepancy was criticised by the interviewees.

### The Swedish legal framework and its application

In Sweden, provisions concerning forest related pathogens, plant and tree species are distributed across different laws and involve several types of legislation; the legal instruments range from performance obligations to species-specific regulations. The lack of comprehensive regulatory framework for the control of invasive alien species was also noted in the *National strategy and action plan for alien species and genotypes*, in which the Environmental Protection Agency called for higher priority of the issue (SEPA 2008). In a 2014 revision of the action plan, the Agency suggests how Sweden can meet the requirements of the EU Regulation on IAS, including the development of an updated national (black)list in addition to the NOBANIS database (SEPA 2014).

The Swedish framework within which new regulation could be developed is largely set by general environmental legislation (the Environmental Code^4^) and sectorial legislation (the Forestry Act^5^ and the Plant Protection Act^6^). As the overarching environmental legislation in Sweden, the Environmental Code is basically applicable to all environmentally related activities. Together with a sustainability objective, the heart of the Code is *the general consideration rules*. These rules reflect all significant environmental legal principles, including the precautionary principle and the polluter pays principle, and are applied mainly in connection with licensing (e.g. permit assessment). The implication of the precautionary principle—as it is expressed here—in the context of IAS is that, while scientific evidence that the species is harmful might be lacking, precautionary measures must be undertaken if there is a risk of harm (Pettersson and Keskitalo 2012). It is further the responsibility of the operator to investigate if and how the operation impacts the environment, e.g. by IAS introduction. The obligation to take precautionary measures also reflects the polluter pays principle; to prevent and counteract damage is also a way to internalise the externalities of the activity. Besides the general environmental requirements and principles, the Environmental Code also specifically controls some issues of relevance for forest-related pathogens and invasive plants and tree species, for instance in accordance with CITES and the Birds and Habitat Directives. Restrictions regarding deliberate release (including planting) of invasive species can thus be issued if necessary to e.g. protect biodiversity (Prop. 1997/98:45). The handling of animals and plants, including import, export, transport and storing, is controlled via government regulations and may include prohibition of certain activities as well as permit requirements. Plant protection products and biocide products containing nematodes, insects or spider animals, which are not subject to EU law, are also governed nationally via ordinances to the Environmental Code.^7^

When asked to what extent they apply the rules of the Environmental Code, the interviewees from the Board of Agriculture answered that they do not apply it regarding plant health, since they have more detailed regulations. The interviewees from the Forest Agency answered similarly, as they have more detailed regulations too. It was however suggested that the Code should be applied to a larger extent, for example in matters relating to environmental impacts of forest seeding and planting materials. The interviewee from the Environmental Protection Agency argued that it is not possible to prevent introduction of IAS with support of the Environmental Code, but that this could be changed.

Some of these limitations are a result of the general principles for the application of law, in this case the principle of *lex specialis*, which entails that if an issue is regulated in special law it supersedes the provisions under general law, i.e. the Environmental Code. Since the Forestry Act has its own consideration rules, this has important consequences for the control of IAS, for example regarding the application of the precautionary principle.

The Swedish forest legislation allows for regulations on the use of forest reproductive material in the establishment of new forest stands if warranted from a silvicultural point of view. Consequently, forest material from outside of the EU may not be introduced in Sweden without permit. A permit may in turn only be granted if the admission is in compliance with Directive 1999/105/EC. The trading of forest material within the EU is also subject to control with regard to invasive species. Certain types of wood and wood from certain areas of origin require a plant passport to ensure that it is free from plant pests. In addition, foreign tree species may only be used as forest reproductive material in exceptional cases, although it is generally allowed to grow the *Pinus contorta* in certain parts of the country.^8^ Thus, the Forestry Act and related legislation focuses more on business as usual, with only limited inclusion of areas related to invasive species. In order to provide possibilities for taking more extensive measures against IAS, it was suggested by the interviewees from the Forest Agency that the Forestry Act should be expanded to include more insect pests, for example via the “control area” instrument.

Finally, sectorial legislation in terms of the Plant Protection Act and regulations of the Board of Agriculture specifically regulate the measures that can be taken to control or hinder the spread of specified plant pests. These are quite detailed and include: action to combat plant pests by property owners; decontamination of e.g. facilities and objects; regulations on cultivation or harvesting; and prohibition or conditions for the handling of plants, plant products, pests, soil etc., including import, export or possession. In addition, regulations for handling of plant pests and decisions on examination of plants, plant products, soil, facilities etc. may be issued to control the spread of plant pests, and to identify the presence of and establish the absence of such pests. To this effect, regulations on health certificates, such as plant passports and labelling in accordance with ISPM 15, may be issued (Prop. 2012/13:174). Regulations also exist for heat treatment, kiln drying and marketing of sawn wood, wood packaging material etc.^9^ Any suspicions that plants or plant crops have been infested with pests must moreover be reported to the competent authority.

The legal framework for plant protection was considered by the interviewees to hold important tools for preventing introduction and spread of plant pests, and to provide extensive powers for taking measures against plant pests. Changes to this system were however anticipated with the new EU Regulation on plant health.

To extend the capacities to act on invasive plant species beyond what is possible under the existing legal framework, it was suggested by several interviewees that Sweden should implement a *risk assessment function*. Currently, Sweden has no capacity to carry out risk assessments compliant with international standards, and more resources were thus considered necessary. A new risk assessment organisation, located at the Swedish University of Agricultural Sciences, has furthermore been proposed by the Board of Agriculture (Swedish Board of Agriculture 2014). The lack of resources was mentioned as a general problem in relation to the work with IAS; too few people are working on the issue, so it progresses slowly, and while the authorities have responsibilities they do not have sufficient resources.

## Discussion and conclusion

The situation in Sweden regarding the legal control of IAS exhibits most of the problems pointed out by Shine et al. (2000): related rules and regulations are spread across different legal areas, including the Environmental Code and other environmentally related acts, like the Forestry Act and its regulations, with a lack of both coordination and coherence as a result (see also Keskitalo and Pettersson 2016; Klapwijk et al. 2016). Plant pests entering new territories as a result of international trade often meet the criteria for a negative external effect; while the influx of pests is a consequence of the trade, the “producer” of the externality has no incentive of taking it into account in the decision making. This is especially the case if the design of the sectoral legislation implies that the Environmental Code and hence the precautionary principle does not apply. To deal with the problem, the external effects must be internalised. I.e. incentives for the operator to include also this aspect on the cost side of the activity must be created for example through a precautionary approach. Under such an approach the operator (who has the most knowledge of the activity) has an extensive obligation to assess the risks of the activity in advance, and take the necessary precautions regarding materials, transport and other protective measures. The large uncertainty present in relation to e.g. the dispersion of damage and the knowledge of factual harm, as well as regarding who is responsible for the damage clearly indicates that the most cost effective way of handling the issue of invasive species is by preventive measures, i.e. *ex ante* (e.g. Pettersson and Keskitalo 2012). While this calls for governance system based on the fundamental principles of caution and polluter pays, regardless of whether they are implemented by law or via other policy instruments (e.g. taxes and fees), the current international trade-based framework exhibits a much less flexible approach. Given the Swedish implementation of the polluter pays principle—which has so far been little emphasised in this regard—the possibilities to impose binding obligations in terms of precautions and requirements in connection with e.g. trade in plants and plant products is relatively large. The Swedish interpretation of the precautionary principle also includes two very important aspects: precautionary measures must be taken already when there is risk of harm, and to avoid the requirements, the operator must show that there is no risk (Michanek 2007). This strong interpretation, if it was applied to invasive species, would conflict with the weak interpretation in the SPS Agreements. As a result of the general acceptance of the weak interpretation, neither Sweden nor the EU can however act independently to protect themselves from the influx of species resulting from international trade (SEPA 2014:20). Therefore, the Swedish Environmental Protection Agency has called for clarification of meaning of the principle and the different standards accepted by the SPS Agreement in order to investigate the possibilities of applying the precautionary principle in the management of invasive species in Sweden (SEPA 2008). However, to include the precautionary principle more strongly would also pose a challenge to what are so far largely systems focused on highly specific and evidenced harm (Harremoës et al. 2001).

## Footnotes

1 The Convention on Biological Diversity (CBD). 2010. Aichi biodiversity targets of the strategic plan 2011–2020. http://www.cbd.int/sp/targets/. Accessed 31 August 2015.

2 Also referred to as a constructivist approach (e.g. Shaffer and Pollack 2010).

3 There are also other agreements that are relevant for the control of IAS, but in terms of the legal effect of the instruments these are secondary in relation to the WTO regime (cf. Perrings et al. 2010).

4 Miljöbalk SFS 1998:808.

5 Skogsvårdslag SFS 1979:429.

6 Växtskyddslag SFS 1972:318.

7 Ordinance (2000:338) on biocide products and Ordinance (2006:1010) on plant protection products.

8 Regulations from the Swedish Forest Agency, SKSFS 1993:2; SKSFS 2010:2.

9 Regulations of the Swedish Board of Agriculture, SJVFS 2004:53.
